# Hippocampal remapping as hidden state inference

**DOI:** 10.7554/eLife.51140

**Published:** 2020-06-09

**Authors:** Honi Sanders, Matthew A Wilson, Samuel J Gershman

**Affiliations:** 1 Center for Brains Minds and Machines, Harvard UniversityCambridgeUnited States; 2 Picower Institute for Learning and Memory and Department of Brain and Cognitive Sciences, Massachusetts Institute of TechnologyCambridgeUnited States; 3 Department of Psychology, Harvard UniversityCambridgeUnited States; University College LondonUnited Kingdom; University of Texas at AustinUnited States

**Keywords:** hippocampus, place cell, context, bayesian inference, hidden state, learning, None

## Abstract

Cells in the hippocampus tuned to spatial location (place cells) typically change their tuning when an animal changes context, a phenomenon known as remapping. A fundamental challenge to understanding remapping is the fact that what counts as a ‘‘context change’’ has never been precisely defined. Furthermore, different remapping phenomena have been classified on the basis of how much the tuning changes after different types and degrees of context change, but the relationship between these variables is not clear. We address these ambiguities by formalizing remapping in terms of hidden state inference. According to this view, remapping does not directly reflect objective, observable properties of the environment, but rather subjective beliefs about the hidden state of the environment. We show how the hidden state framework can resolve a number of puzzles about the nature of remapping.

## Introduction

Place cells of the hippocampus fire when an animal occupies specific spatial locations (place fields; O'Keefe, 1976). Each place cell has its own respective place fields, so collectively the population of place cell comprise a map of an environment, in which each location corresponds to activity of a particular subset of place cells. The hippocampus is thought to use independent maps for each context. These independent maps can be observed through ‘‘place field remapping’’, in which the location of a place field may change or the place field may disappear entirely between contexts (Muller and Kubie, 1987; Colgin et al., 2008; Kubie et al., 2019). The sensitivity of place cells to context changes is consistent with many other studies implicating the hippocampus in context-dependent behavior (Holland and Bouton, 1999; Gershman et al., 2010; Anagnostaras et al., 2001; Smith and Mizumori, 2006a). Despite its acknowledged importance, the precise relationship between context changes and remapping has remained elusive, due in part to ambiguity as to what counts as context change.

Researchers have operationalized context in many different ways. For example, some researchers investigated the role of sensory cues (Knierim et al., 1998; O’Keefe and Conway, 1978; Muller and Kubie, 1987), whereas others investigated the effect of changing spatial location or geometry (Skaggs and McNaughton, 1998; Lever et al., 2002), or changing the task (O'Keefe and Speakman, 1987; Markus et al., 1995). Not surprisingly, different effects have been observed for these different manipulations, without cohering into a unified picture of how context changes determine remapping.

Some of the confusion about what counts as a context change is due to inconsistent definitions of the word 'context'. Sometimes 'context' refers to experimenter-defined variables, such as physical location or sensory cues. In other cases, 'context' refers to the animal’s internal assessment of the environment as indicated by neural activity or behavioral response. For example, in the fear conditioning literature, animals are assumed to preferentially freeze in the 'same' context as that in which they received the shock. This doesn’t necessarily have to be physically the same environment, as long as the animal infers that it is the same environment (Chang and Liang, 2017; Gershman et al., 2010). Invoking subjective inferential factors in the interpretation of remapping compels us to consider basic questions about the nature of these inferences. What is the animal’s hypothesis space? How does it represent and update beliefs over this hypothesis space?

The goal of this paper is to develop formal answers to these questions, and thereby provide a coherent account of diverse experimental findings. Key to this account is the idea that the relationship between observable properties of the environment (including context) and remapping is mediated by inferences about unobservable properties of the environment (*hidden states*). We emphasize for clarity that the ‘‘observable’’ properties of the environment have themselves been inferred through sensory processing and therefore are in a sense hidden, but when we refer to hidden states, we are referring to regularities in the environment that could not be observed even with perfect sensory reproduction of the environment. According to this view (see also Fuhs and Touretzky, 2007; Gershman et al., 2014; Penny et al., 2013), place fields remap when the animal believes that it has entered a new hidden state. By specifying the animal’s internal model of how hidden states relate to observable stimuli, we can make principled predictions about when, why and how place fields remap.

Before describing the details and applications of this computational framework, we will briefly review some of the key empirical and theoretical background.

### Empirical background

Remapping phenomena have been divided into several classes (Colgin et al., 2008; Muller, 1996; Kubie et al., 2019). At the extremes, there is ‘global’ or ‘complete’ remapping (where no place fields are shared between contexts) and ‘null’ or ‘lack of’ remapping (where all place fields are shared between contexts). Between these extremes is ‘partial remapping’ (where some place fields are shared between contexts but some are not) and ‘rate remapping’ (where place fields are shared between contexts but have characteristically different firing rates). However, none of these categories can be regarded as strictly exclusive.

The extent to which place fields are shared between contexts can be quantified by looking at the spatial correlations of place cell firing rates between contexts. Although studies report correlations near zero between place fields in different contexts (Leutgeb et al., 2004; Muller and Kubie, 1987; Schlesiger et al., 2015), there are reasons to believe that correlations are not actually zero. A recent report suggests that previous observations of global remapping might be artifacts of misalignment of maps between contexts (Kinsky et al., 2018). Some place cells have been found to consistently encode reward across virtual reality contexts that otherwise express ‘global remapping’ (Gauthier and Tank, 2018), so there is at least one class of place cells that have recently been found not to remap across contexts. More generally, many studies reporting global remapping report low but non-zero correlations (Leutgeb et al., 2004; Skaggs and McNaughton, 1998; Spiers et al., 2015).

Conversely, studies reporting lack of remapping never report perfect place field overlap between contexts. Indeed, even within a single context, patterns of spatial firing show variability over time, as if more than a single map is used in a given context (Fenton and Muller, 1998; Kay et al., 2019; Kelemen and Fenton, 2016). Additionally, the extent of remapping for repeated presentations of the same context depends on the amount of experience the animal has had (Law et al., 2016).

Rate remapping is also not a strict category. Manipulations used to generate rate remapping do so for a fraction of the place cell population, while other cells in the population maintain or lose their place fields (Wood et al., 2000; Leutgeb et al., 2005a). In this way, rate remapping is always accompanied by partial remapping. Additionally, protocols for generating rate remapping can sometimes produce a range of remapping states during learning, ranging from no remapping to global remapping. For example, Leutgeb et al., 2005a found rate remapping when comparing place field maps between circle and square enclosures. However, Lever et al., 2002 make the same comparison between circle and square enclosures, and find rate remapping as an intermediate state as the animal transitions from no remapping to global remapping over the course of learning.

The complications discussed above highlight the fact that virtually all remapping is partial remapping. Place cell responses to manipulations are extremely heterogeneous (Lee et al., 2004; Shapiro et al., 1997; Chen et al., 2013; Anderson and Jeffery, 2003). Additionally, remapping behavior can vary across animals (Wills et al., 2005; Lever et al., 2002) as well as across laboratories (Guzowski et al., 2004; Wills et al., 2005; Leutgeb et al., 2005b; Colgin et al., 2010), see the ‘‘Morph Experiments’’ section of the Results for an in-depth exploration of one example). We will argue that this heterogeneity arises from variability in beliefs across animals.

### Theoretical background

Our theory of hidden state inference is motivated by, and builds upon, prior research into the nature of context-dependent learning. Since Pavlov, experimentalists have recognized that extinguishing an association after Pavlovian conditioning is not the same as unlearning it. The association can return under a variety of circumstances (Bouton, 2004), such as returning the animal to the conditioning context, or simply waiting a period of time before testing the animal. These phenomena seem to suggest that the animal is forming a new memory during extinction, which could compete with the conditioning memory at the time of retrieval. Context, on this view, serves as a particularly powerful retrieval cue. The fundamental challenge posed by this interpretation is to define precisely the conditions under which a new memory is formed or an old memory is updated, and the conditions under which a particular memory is retrieved at the time of test.

One approach to these questions is to frame them in terms of hidden state inference (Gershman et al., 2010; Gershman et al., 2017a): new memories are formed when an animal has inferred that it has encountered an unfamiliar (previously unvisited) state, and old memories are updated when it has inferred that it has encountered a familiar state. As we formalize below, these inferences can be calculated using Bayes’ rule, which computes a posterior probability distribution over hidden states by integrating prior beliefs about the hidden states with the likelihood of those hidden states given the animal’s observations. The hidden states are sometimes interpreted as *latent causes* (Courville et al., 2006; Gershman and Niv, 2012b), to emphasize the idea that the animal is forming beliefs about the causal structure of the environment.

The state inference framework can naturally explain many animal learning phenomena (see Gershman et al., 2015), for a review). For example, a conditioned response takes longer to extinguish when reward is delivered probabilistically during the acquisition phase, a phenomenon known as the *partial reinforcement extinction effect* (e.g., Gibbon et al., 1980). This phenomenon is surprising for classical associative learning accounts, since the learned association should be weaker under partial reinforcement, and hence should be *faster* to extinguish. According to the state inference framework, partial reinforcement renders the hidden state ambiguous; it takes more extinction trials until the animal is confident that acquisition and extinction trials were generated by different states (Courville et al., 2006; Gershman and Blei, 2012a).

In this paper, we argue that the same framework can unify many different place field remapping phenomena, under the assumptions that (i) each map corresponds to a unique hidden state, and (ii) a map is activated in proportion to the posterior probability of the corresponding hidden state. A closely related idea was pursued by Fuhs and Touretzky, 2007, to which we owe the inspiration for the present work. Our goal is to explain a significantly broader range of phenomena using a somewhat simpler model, and to resolve a number of lingering empirical puzzles. In particular, we stress the role of uncertainty in hidden state inference and its connection with partial remapping, rate remapping, and population heterogeneity. This connection allows us to explain phenomena such as the stabilization of place cell maps over time and the potential role of experience in place cell responses to morph enclosures, among other phenomena.

## Results

### Conceptual overview of the model

The computational problem facing the animal is to infer the posterior probability of each hidden state c given its observations 𝐲 (e.g., geometric or color features of a box), as stipulated by Bayes’ rule:

(1) P(c|y)∝P(y|c)P(c),

where P⁢(𝐲|c) is the likelihood of the observations under the hypothetical state c, and P⁢(c) is the prior probability of state c. A more detailed formal description of these terms can be found in the Materials and methods. In this section, we describe intuitively what they mean and how they work.

**Figure 1.**
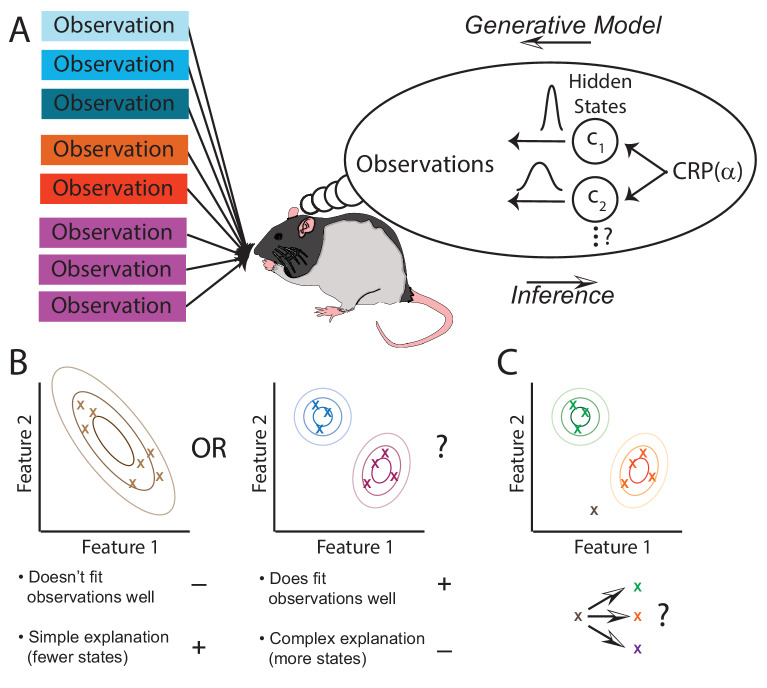
The hidden state inference framework. (**A**) Schematic of hidden state inference. We impute an internal generative model to the animal, according to which observations are generated by a small number of hidden states. States are sampled from the Chinese Restaurant Process, parametrized by α (see Materials and methods for details). Each state is associated with a particular distribution over observations. The animal receives those observations but does not have direct access to the states that generated them. We model the animal as probabilistically inverting this generative model by computing the posterior distribution over hidden states given observations. (**B**) Example inference problem. Given a set of observations (x’s), the animal must infer how many hidden states there are. There is a tradeoff between increasing the number of hidden states in order to better fit the observations vs. decreasing the number of states in order to decrease the complexity of the explanation. The partition evidence ratio can be calculated given a particular set of observations to express the relative preference for the 1-state model vs. the 2-state model. See Equation 7 in the Materials and methods section for more details. (**C**) Another example inference problem. Given an assignment of past observations (green and orange x’s) to hidden states (green and orange) and a novel observation (gray x), the animal forms a belief about hidden state assignment of the novel observation. This belief consists of probabilities of assigning the novel observation to each of the past hidden states (green or orange) or alternatively to a novel hidden state (purple). We can compare any two of these alternatives with the state evidence ratio. See Equation 11 in the Materials and methods section for more details.

The animal is presented with observations that are generated by an unknown number of states through a process that the animal is not aware of (left side of Figure 1A). The animal builds an *internal model* of the world (thought bubble in Figure 1A). That model doesn’t have to mimic the world exactly, it simply needs to be flexible enough to be able to capture the structure that it is presented with. We suggest that the animal’s internal model provides a generative ‘‘recipe’’ through which it assumes observations are produced: first a state is sampled from P⁢(c), and then an observation is sampled from the distribution associated with that state P⁢(𝐲|c). The job of the animal is to invert this generative process and infer the posterior probability of each hidden state c given its observations 𝐲. Since different states could theoretically produce the same observations, the animal is faced with fundamental ambiguity. The posterior distribution P⁢(c|𝐲) represents the animal’s uncertainty about the hidden state. As it collects more observations and thereby reduces its uncertainty, the posterior will tend to progressively concentrate on a single explanation of which observations come from which states.

Because there is no reason to assume that the animal has a priori knowledge about the set of states, we allow the state space to potentially grow as the animal collects new observations. The animal starts off with a single state, and at each new observation it can assign some probability to a new state or one of its previously inferred states. As detailed in the Materials and methods, we accomplish this using a Bayesian nonparametric prior over hidden states. Importantly, this prior favors a small number of hidden states, encoding a form of ‘simplicity bias' or Occam’s razor.

As mentioned in the Introduction, we assume a one-to-one correspondence between hidden states and maps. Thus, we transpose the question ‘did the place field remap?’ to ‘were these observations generated by the same hidden state?’ More precisely, we report the log posterior probability ratio between 1-state and 2-state hypotheses (or *evidence ratio*, for brevity), which we take to be related to the degree of remapping (see Materials and methods for definitions of two versions of the evidence ratio: the partition evidence ratio and the state evidence ratio). When the evidence ratio is near 0, the animal is indifferent between the two hypotheses, and in this case we expect partial remapping. No remapping occurs when the evidence ratio is strongly positive (favoring the 1-state hypothesis), rate remapping occurs when the log probability ratio is weakly positive, and global remapping occurs when it is strongly negative. Keep in mind, following our overview of the literature in the Introduction, that these are heuristic categories without strict boundaries. On the probabilistic view, these categories occupy different points along a spectrum.

### The effect of sensory cues

One of the first questions asked about hippocampal remapping was which sensory cue controls whether a map is used. The first study of remapping O’Keefe and Conway, 1978 found that in an environment with four cues, some place fields disappeared with the removal of one or two cues, but most place fields maintained their firing with the removal of any two cues. In more modern terms, removal of a subset of cues caused partial remapping, but there was not a one-to-one correspondence between place fields and cues. Thus, from the very beginning it was clear that remapping is not in response to cues but in response to cue constellations (see also Shapiro et al., 1997; Fenton et al., 2000; Muller and Kubie, 1987). Each of these studies involved separately rotating or removing groups of stimuli, finding that many place fields that rotated when a given stimuli was rotated still maintained their firing when that stimuli was removed. A similar early result was that of O'Keefe and Speakman, 1987, where cues necessary for orientation of the map were removed, but the place cell map was maintained. The significance of these results is that the place field map is responsive to cues but is not controlled by cues in a one-to-one fashion.

Viewing remapping as hidden state inference provides an important insight into this behavior. Our model posits that the cues jointly inform the posterior over hidden states. Individual cues will typically only exert a weak effect on the posterior, and hence exert only a weak effect on remapping.

**Figure 2.**
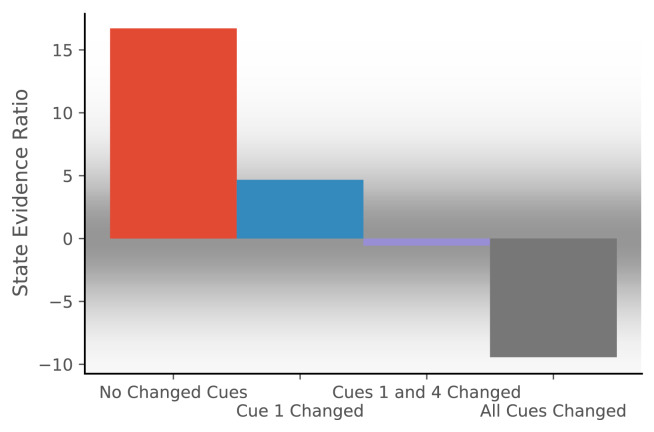
Hidden state inference is informed by cue constellations. Observations are generated from a distribution with four features, each drawn from a Gaussian with mean 0 and standard deviation of 0.2.We train the model with 20 observations drawn from that distribution. We then compare the posterior probability of assigning a probe observation to the same hidden state as the previous observations vs. assigning it to a novel hidden state (Equation 11 for same c vs. novel c). The first probe is an observation where each feature has a value of 0 (no cues changed). The model prefers assigning this probe observation to the same hidden state as the previous observations, corresponding to no remapping. The second probe is an observation where the first feature has a value of 1 and the other features have values of 0 (cue one changed). The third probe is an observation where the first and last features have a value of 1 and the other features have values of 0 (cues 1 and 4 changed). For both of these, the model assigns a state evidence ratio near 0, representing relatively high uncertainty about hidden state assignment, which corresponds to partial remapping. The grey background has saturation proportional to a Gaussian centered at 0 with a standard deviation of 5; values with a grey background can be heuristically thought of as partial remapping, whereas values with a white background can be thought of as either complete remapping or lack of remapping depending on whether two states are more likely (negative values) or one state is more likely (positive values). The fourth probe is an observation where all four features have values of 1 (all cues changed), for which the model prefers assigning the probe observation to a new hidden state, corresponding to global remapping.

To simulate the effect of cue configurations on remapping, we assume that the observation vector consists of four features, each drawn from a Gaussian with mean 0 and standard deviation of 0.2. We provide the model with 20 observations drawn from that distribution and then provide one of four probe observations. For each probe, we compute the state evidence ratio (Figure 2).

The first probe is an observation where each feature has a value of 0 (no cues changed). The model prefers assigning the probe observation to the same hidden state as the previous observations, corresponding to no remapping. The second probe is an observation where the first feature has a value of 1 and the other features have a value of 0 (cue 1 changed). The third probe is an observation where the first and last features have a value of 1 and the other features have a value of 0 (cues 1 and 4 changed). For both of these, the model produces an evidence ratio near 0, registering a high level of uncertainty about the hidden state (i.e., partial remapping). The fourth probe is an observation where all four features have a value of 1 (all cues changed), for which the model prefers assigning the probe observation to a new hidden state, corresponding to global remapping. These simulations demonstrate how the model is sensitive to the configuration of cues; no one cue completely controls remapping, consistent with the experimental data reviewed above.

Another aspect of these simulations worth highlighting is the fact that they are probabilistic. The representation of uncertainty in hidden state identity corresponds in an important way with the result that hippocampal maps during two experiences are almost never entirely overlapping nor entirely independent. From the perspective of our model, this ‘partial remapping’ reflects the inherent uncertainty about whether different observations are drawn from the same distribution.

### Experience-dependent remapping

**Figure 3.**
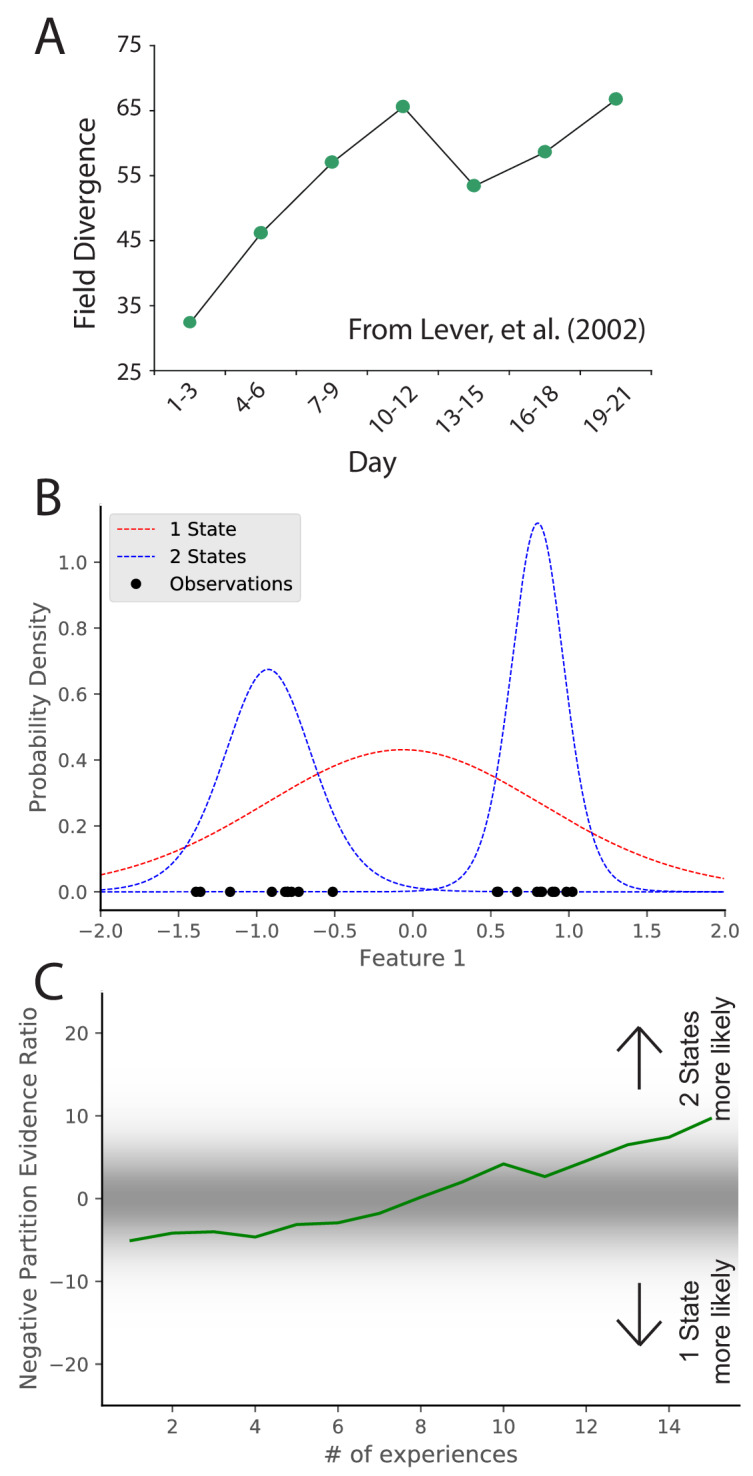
Learning to distinguish. (**A**) Adapted from Lever et al., 2002, who compared place cell representations between alternating presentations of square and circle boxes. Field Divergence is expressed in percent and represents the fraction of place fields that remap between the two enclosures. The representations of the enclosures are initially similar, but diverge with learning. (**B**) Simulated observations (black dots) are generated from Gaussians centered at −1, 1. The model compares the posterior probability of the observations coming from one inferred hidden state (red) or two inferred hidden states (blue). (**C**) The relative probability assigned to the observations coming from two hidden states vs. one hidden state (Equation 7) is shown as a function of amount of experience. Early on, there is uncertainty about how many hidden states there are, whereas later two hidden states is more probable, similar to the empirical observations. As in Figure 2, values with a grey background can be thought of as partial remapping whereas values with a white background can be thought of as either complete remapping or lack of remapping depending on whether two states are more likely or one state is more likely. Note that the axis here has been flipped relative to Figure 2 in order to match the axis of the empirical results shown in panel A.

The previous section addressed the study of how sensory cues control place field remapping. Another line of research has studied how more diffuse contextual cues control remapping, but the answer was invariably that it depended on prior experience (Knierim et al., 1995; Sharp et al., 1990; O'Keefe and Speakman, 1987; Breese et al., 1989; Knierim et al., 1998; Bostock et al., 1991; Shapiro et al., 1997). One prime example of this is the role of environmental geometry (the shape of the recording arena). Initially, it was thought that different geometries necessarily corresponded to different maps (Muller and Kubie, 1987; Quirk et al., 1992) , but recordings had always been done in familiar environments. The first group to record throughout the course of learning found that there was no consistent relationship between environment shape and inferred hidden state (Lever et al., 2002). In this experiment, place cells were recorded in rats who were alternately placed in square and circle boxes occupying the same location in the recording room day after day. Early in learning, there was limited remapping. Only after extensive experience in the two boxes did the animals remap between the two boxes (Figure 3A). This indicates that the sensitivity to context changes changes with experience. Analogous results have been found for the effects of experience on remapping in response to other manipulations (Bostock et al., 1991; Shapiro et al., 1997). These effects are hard to explain in terms of fixed contextual boundaries governing remapping. It is naturally explained by the hidden state inference perspective, which posits that uncertainty about hidden states evolves as more data are observed. In particular, distinctions between hidden states are acquired gradually, such that substantial remapping should only be observed after sufficient experience to counteract the ‘‘simplicity bias’’ favoring a small number of hidden states.

We simulate these experiments qualitatively in the following way. We take observations to be 1D for simplicity, where the single dimension is the feature along which the distinction is learned. For example, in the circle-square experiment (Lever et al., 2002), the dimension would be the shape of the enclosure. We generate observations from two Gaussians (corresponding to the circle and square contexts) with $\mu_{1}=-1,\mu_{2}=1,\sigma_{1}=\sigma_{2}=0.3$ (Figure 3B). We alternate drawing observations from each distribution. After each pair of draws, we compute the partition evidence ratio (in this case, the relative probability of the hypothesis that all observations up to that point were drawn from a single hidden state against the hypothesis that all observations up to that point had been drawn from two alternating hidden states).

Early in training, there is uncertainty about how many hidden states there are (Figure 3C); the evidence provided by the observations is not yet sufficiently strong to overwhelm the simplicity bias of the prior. As more data are observed, the two-state hypothesis is eventually favored over the one-state hypothesis. The hidden state inference perspective thus explains why context-dependent remapping only emerges gradually with experience.

### Stabilization of maps over time

**Figure 4.**
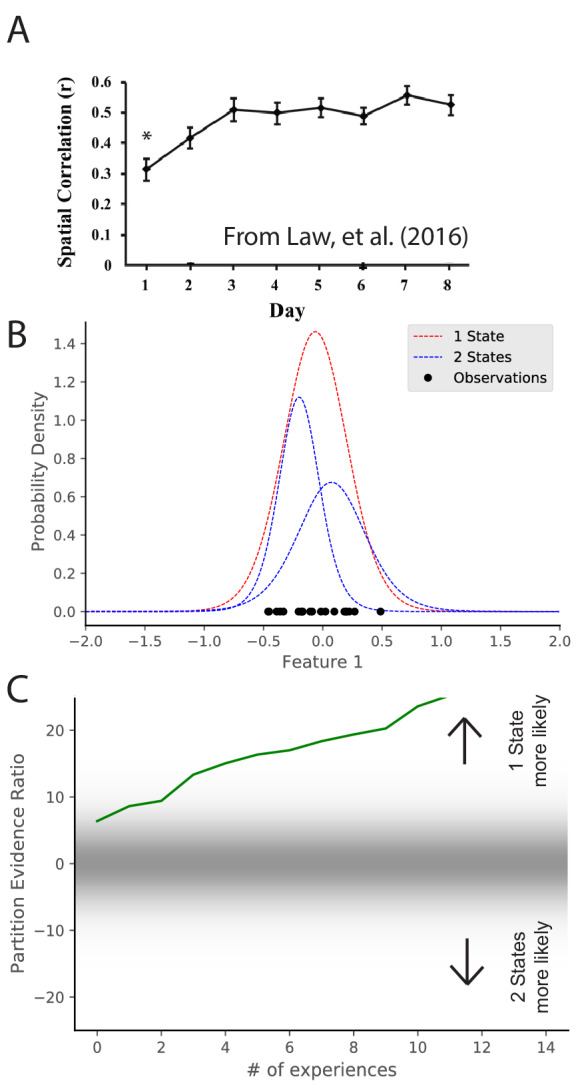
Map stabilization requires certainty about distributional statistics. (**A**) Data from Law et al., 2016, showing the spatial correlation of the hippocampal map in repeated presentations of the same environment over multiple training days. Initially, the correlation is low, indicating extensive remapping between observations, but over the course of training the extent of remapping between observations decreases. (**B**) Observations (black dots) are generated from two Gaussians, both of which are centered at 0. The model compares the posterior probability of the observations coming from one inferred hidden state (red) or two inferred hidden states (blue). (**C**) The relative probability assigned to the observations coming from one hidden state vs. two hidden states (Equation 7) is shown as a function of amount of experience. Early in training, the two hypotheses have similar probabilities, whereas later one hidden state is overwhelmingly more probable. This corresponds to an increase in certainty over training, which would translate into a decreased tendency to remap, similar to the empirical observations. Note that the axis here has been flipped relative to Figure 3C in order to match the axis of the empirical results shown in panel A.

Maps take time to stabilize: repetition of a novel environment induces less map similarity than repetitions of a familiar environment (Frank et al., 2004; Leutgeb et al., 2004; Law et al., 2016). In particular, Law et al., 2016 alternated presentation of two environments. They found that intra-environment map similarity went up as a function of experience (Figure 4A). These results are difficult to explain under the assumption that remapping is induced by the discrepancy between expectations and current cues exceeding a fixed threshold (Jeffery, 2003). Long-term potentiation (LTP) had been tied to map stabilization (Kentros et al., 1998; Cobar et al., 2017), but the speed with which LTP can create place fields (single trials; Bittner et al., 2017) is inconsistent with the slowness of map stabilization. The hidden state inference perspective offers a different interpretation of map stabilization: as an animal gains more experience with a particular state, it sharpens its representation of that state (i.e., its uncertainty about the distributional statistics decreases), and consequently it becomes more confident in recognizing repetitions of that state.

We can model the dynamics of stabilization by considering observations which are generated from a single distribution with mean 0. We can consider the same hypotheses as were considered in Figure 3, namely, that there are either 1 or two hidden states. We consider the same hypotheses but the actual generative process has the opposite structure as Figure 3. Through the course of learning, the partition evidence ratio accumulates evidence in favor of the one-state hypothesis, corresponding to the emergence of a ‘‘stable’’ map. Indeed, early in learning, the animal does not know whether it is receiving observations from the simulation of Figure 3 or the simulation of Figure 4, as they are indistinguishable. Only after extensive experience is the animal able to identify which generative process is generating its observations.

### Remapping due to non-sensory changes

**Figure 5.**
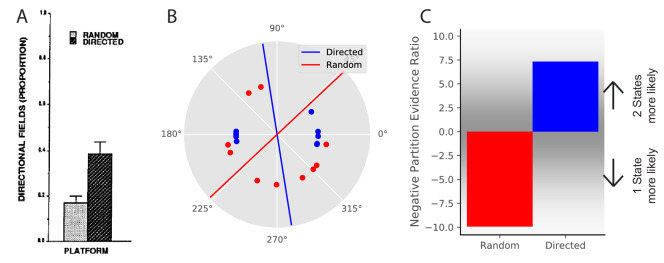
Place field directionality depends on statistics of behavior. (**A**) Data from Markus et al., 1995, showing that place field remapping depends on the animal’s direction more when the animal is running in a stereotyped path than when the animal is running in random directions. (**B**) The model receives circular observations corresponding to the animal’s running direction. The model either receives observations drawn from a uniform distribution (red dots) or alternating from two Von Mises distributions with means of 0 and 180 degrees, and κ=10 (blue dots). These observations are separated into two groups with a line that is the farthest from any observations (red and blue lines). (**C**) The partition evidence ratio between the hypothesis that all observations have been drawn from two hidden states separated by the lines in panel B vs. the hypothesis that all observations have been drawn from a single hidden state (Equation 7) after 10 observations. The model is more likely to put probability on the hypothesis that there are two hidden states when given the directional observations as opposed to the uniform observations. This is similar to the empirical results, where place fields are more likely to remap (more likely to infer two hidden states) when the animal is running in a directed fashion.

Remapping is not solely driven by sensory aspects of experience. For example, place fields can remap depending on internal variables such as movement direction or task (Smith and Mizumori, 2006b; Sanders et al., 2019; Wood et al., 2000; Muller et al., 1994). In general, it is known that place fields can remap depending on which direction the animal is running on a linear track (Markus et al., 1995; Battaglia et al., 2004). However, place fields tend not to remap based on running direction in an open field. This is most clearly shown in Markus et al., 1995. They compared two conditions, both of which occurred in an open field: one in which the animal was randomly foraging, and one in which the animal was running between four specific locations in one of two directions. They found that the extent of remapping in response to movement direction was larger in the directed foraging condition than in the random foraging condition (Figure 5A) despite having the same sensory cues in the two conditions.

From the perspective of hidden state inference, we can draw an analogy with the remapping observed after training in the circle and square boxes (Figure 3), replacing the sensory features of the environment with the non-sensory information about self-motion. In the directed foraging case, observations are clearly separated into two states (clockwise movement and counterclockwise movement), whereas in the random foraging case, there is no consistent partition that could support the inference of multiple states.

We model this experiment in the following way. Again, we take observations to be 1-dimensional for simplicity, where the single feature is the animal’s movement direction. This feature is represented as a circular (angular) variable, as movement direction is circular. We model the random foraging condition as observations drawn from a uniform distribution over the circle (red dots in Figure 5B). We model the directed foraging as observations drawn from a Von Mises distribution with μ=0,κ=10 alternating with a Von Mises distribution with μ=π,κ=10 (blue dots in Figure 5B). For each condition, we separate the observations into two groups with a line for which the distance from any observations is maximum (red and blue lines in Figure 5B). After 10 observations, we ask the model what the relative probability is that the observations were drawn from a single hidden state or drawn from two hidden states split by the line of maximum separation. The model assigns greater probability to the two-state hypothesis for directed foraging. In contrast, it assigns greater probability to the one-state hypothesis for random foraging (Figure 5C). This corresponds to the empirical finding that place fields were more likely to remap under the directed foraging condition compared to the random foraging condition.

### Cue rotation experiments

**Figure 6.**
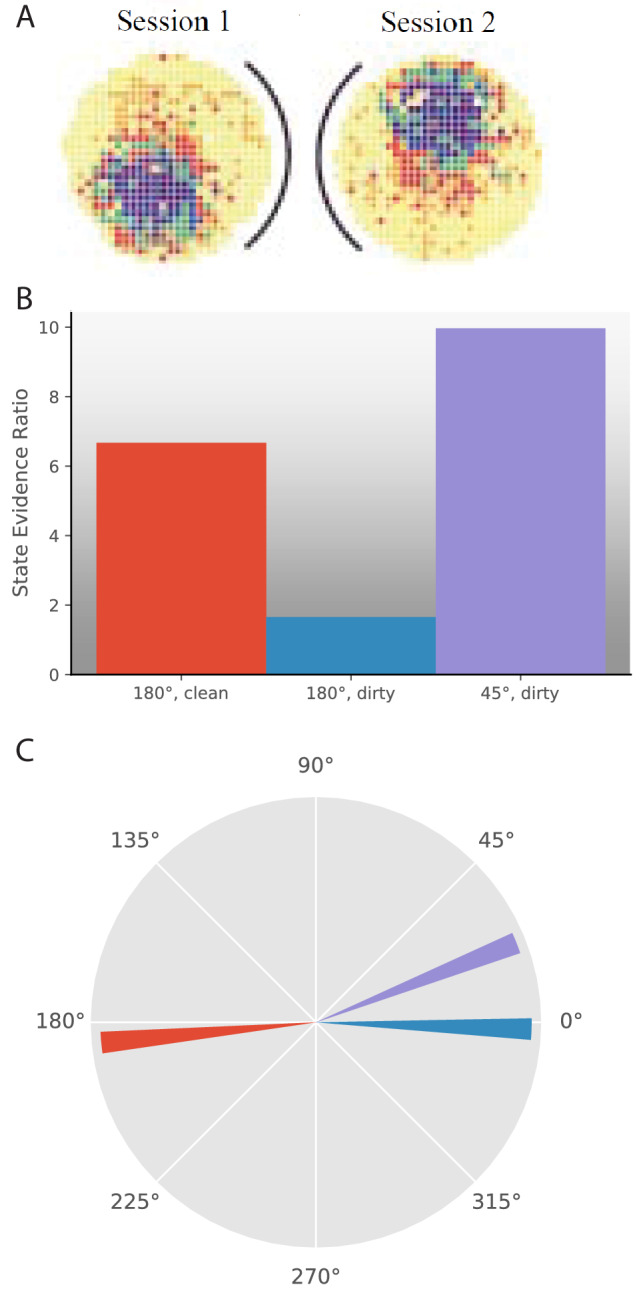
Response to cue rotation depends on experimental protocol. (**A**) Data from Rotenberg and Muller, 1997. The black curve represents the location of the cue card. The heat map represents the firing rate of a given place cell. On rotation of the cue card by 180°, the place field is maintained, but rotated 180° with respect to the room reference frame. (**B–C**) Results of simulation of several experimental manipulations: In ‘180°, clean’, the cue card is rotated 180° while the animal is absent and the maze is cleaned before returning the animal. In ‘180°, dirty’, the cue card is rotated 180° while the animal is present and odor cues left by the animal are not removed. In ‘45°, dirty’, the cue card is rotated 45° while the animal is present and odor cues left by the animal are not removed. (**B**) The state evidence ratio is in favor of assigning to the same hidden state in all three manipulations. However, there is more uncertainty under the ‘180°, dirty’ manipulation. (**C**) The highest probability reference direction is depicted for each manipulation.

One series of experiments used rotation of cues with respect to the recording arena to ask how the place cell representation responds to such changes. The most simple version of these experiments had a circular arena with a cue card on one side of the arena. The cue card could be rotated to any position in the arena, reported as an angle with respect to the original cue card orientation in the room reference frame (Rotenberg and Muller, 1997; Knierim et al., 1998; Hargreaves et al., 2007). Experiments reported two types of changes in place field behavior in response to a given manipulation. One is extent of remapping, as we have been discussing in this paper. The other is which rotational angle the map is oriented towards. This added question is due to the inherent ambiguity in circular variables. Even if a place field moves to a different location in a given reference frame, it is still possible that remapping did not occur if the relative locations of place fields are preserved. Therefore, one must check whether the place field had the same location subject to a rotational offset. This rotational offset frequently corresponds to the rotational offset observed in head direction cells simultaneously recorded from a variety of brain regions (Knierim et al., 1995; Hargreaves et al., 2007). Experimental papers thus report 1) whether place fields remap and 2) if not, whether there is a rotational offset in their locations (Figure 6A).

We model these experiments as follows. Similar to other simulations in this paper, each observation is a feature vector. However, instead of each entry in the vector containing the value of that feature on some sensory axis, the entry contains the angle between a given cue and an uncued direction in the room reference frame. Feature vectors with different values can potentially be identical if there is an offset that can be subtracted from each entry in one vector to give the other vector, corresponding to usage of a different uncued direction as the reference. Therefore, before performing hidden state inference, the animal must decide what reference direction to use in comparing the current observation to past observations from each hidden state. We model reference direction inference in the following way. Given the past observations previously assigned to a given hidden state, we can calculate the offset ϕ to apply to the current observation that gives the maximum value of the posterior predictive distribution (Equation 9) ${argmax}_{\phi }P(y_{t+1}-\phi |Y_{c_{k}})$. Say for example that a certain cue had always been +30° from the reference direction, but this time the cue location is provided as +120°. An offset of −90° would give the maximum probability of generating this observation from the same hidden state. This offset is calculated independently for each hidden state, and the state evidence ratio is calculated using the best offset for each hidden state. The offset of the most likely hidden state would correspond to the rotational offset in the place field locations.

We capture several empirical findings.

The animal is trained with a cue card consistently at 0° with respect to the minimally-cued room reference frame. The animal is removed from the maze, which is cleaned and the cue card is rotated 180°, before returning the animal. The finding is that place fields retain their positions relative to each other (no remapping) and relative to the card, so they rotate 180° with respect to the room reference frame (offset of 180°) (Rotenberg and Muller, 1997; Knierim et al., 1995). We model this by providing 10 single-dimensional training observations, each drawn from a wrapped normal with $\mu =0^{\circ },\sigma =18^{\circ }$, representing the position of the cue card. Then we test with an observation with value 180°. The best offset for the current observation is −175° for the same hidden state as the previous observations (Figure 6C, red). With that offset, the state evidence ratio is in favor of assigning to the same hidden state (Figure 6B, red). Assigning to the same hidden state and 175° offset in the model correspond to the empirical finding of limited remapping and ∼180° rotation of place fields.

A similar experiment was performed where the cue card is rotated 180° without removing the animal or cleaning the maze. The finding is that the place fields did not remap or rotate in response to this manipulation (Rotenberg and Muller, 1997). We model this with an expanded feature vector because the animal has access to additional cues, albeit cues that are less reliable than the cue card: namely, a preserved internal orientation from path integration and odor cues that the animal has left on the maze. The first entry in the feature vector is the same as in the previous simulation, that is the cue card position drawn from a wrapped normal with $\mu =0^{\circ },\sigma =18^{\circ }$. Five additional entries are included in the feature vector with uniformly distributed means and $\sigma =18^{\circ }*3=54^{\circ }$. The larger standard deviations on the positions of these cues correspond to their lower fidelity (Save et al., 2000; Hardcastle et al., 2015). The test observation has a value of 180° for the first entry (cue card) and values of the cue means for the other entries. Our model finds that the best offset is −2° (Figure 6C, blue) and the state evidence ratio is in favor of assigning to the same hidden state (Figure 6B, blue). However, the evidence ratio is much closer to 0, which would predict a larger degree of heterogeneity in place field behavior than the earlier experiment, which is a comparison for which there was not sufficient empirical power (Rotenberg and Muller, 1997). See also Hargreaves et al., 2007, Lee et al., 2004, and Shapiro et al., 1997 for other reports of heterogeneity during cue conflict rotation experiments.

What if the cue card was only moderately rotated in the animal’s presence? Rotenberg and Muller, 1997 rotated the cue card by 45° in the animal’s presence without cleaning the maze. They found that the place fields did not remap and rotated by 45°. We model this experiment the same way as the last experiment except that the test observation has 45° as its first entry. Our model finds that the best offset is 22° (Figure 6C, purple) and the state evidence ratio is in favor of assigning to the same hidden state (Figure 6B, purple). The difference between the results of this and the last experiment is due to the fact that the other cues have large enough variance to accommodate a 45° rotation without requiring a new hidden state.

To summarize this section, rotation experiments share a framework with other cue manipulation experiments with the added complication of estimation of the appropriate rotational reference direction. It is therefore possible for place fields to retain their relative arrangement while also rotating with respect to some reference frame, which we consider to be a lack of remapping (assignment to the same hidden state). If the posterior probability of an observation is sufficiently low even after picking the best rotational reference, then a new hidden state would be inferred and place fields would lose their relative arrangement.

### Morph experiments

A persistent puzzle in the field is the inconsistent results from ‘morph’ environments that interpolate between different geometries (e.g., square and circle). Different labs have found different results with experimental setups that are not directly comparable (Wills et al., 2005; Leutgeb et al., 2005b; Colgin et al., 2010). We summarize the past results here and suggest an interpretation that leads to a novel prediction.

**Figure 7.**
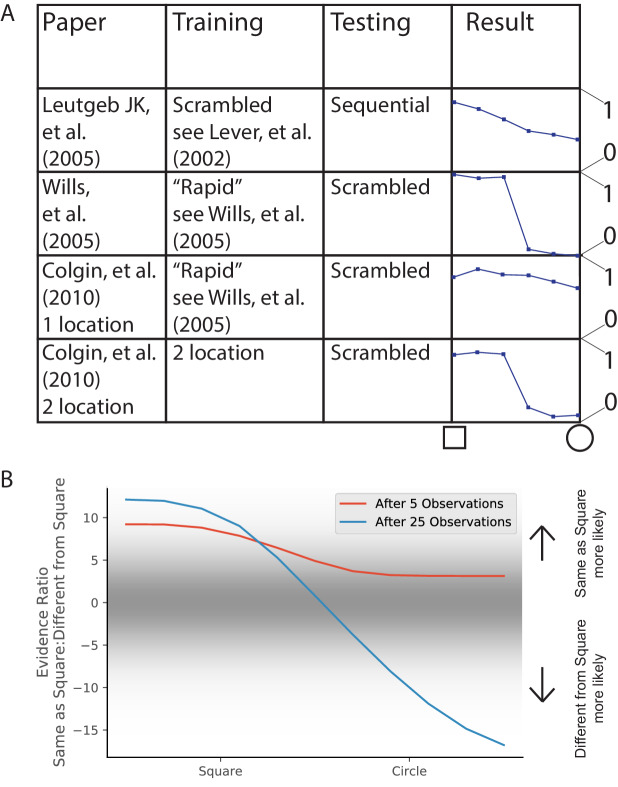
Morph experiments. (**A**) Different experimental protocols give different results for the morph experiment. The results in the fourth column show the similarities in population representation of the intermediate morph shapes compared to the square shape. The results in the fourth column are adapted from the corresponding paper cited in the first column. All values are shown on a scale ranging from 0 to 1, where one is complete concordance of population representations and 0 is random concordance. We classify the results into two qualitative classes: the first and third rows have results where all levels of morph result in partial remapping, whereas the second and fourth rows switch between no remapping and complete remapping as morph level increases. Scrambling during testing does not seem to be related to this effect. Moreover, the same experimental protocol can have qualitatively different results in different labs (compare second and third rows). (**B**) We provide observations from two alternating Gaussians with means −1 and +1, just as in Figure 3. We test after 5 (red) or 25 (blue) training observations by providing intermediate values and measuring the relative probability of being assigned to the same hidden state as the −1 mean observations. We thus predict that both qualitative results can be achieved in the same lab simply by performing the morph testing at different points of training.

In 2005, two groups each performed an experiment to answer the question, ‘How does the hippocampus represent a novel environment that is intermediate between two familiar environments?’ Both groups familiarized rats in square and circle environments, and then tested them in intermediate environments (polygons with a variable number of sides). The two papers had different results (Figure 7A), characterized at the time in terms of whether the similarity curve had a discrete switch or a gradual switch. However, this difference is extremely hard to robustly characterize, considering that the variation in similarity between repetitions of the same environment was half as large as the entire range of similarity variations for the entire morph sequence (compare first and last points in Leutgeb et al., 2005b, their Figure 6E). The other difference that was discussed at the time was whether the population response was coherent or heterogeneous. While both studies showed heterogeneous population responses, they did show different levels of heterogeneity, and we discuss this in the next Results section (Population heterogeneity and rate remapping).

A much more striking point of comparison was the difference in the extent of remapping between the extreme square and circle environments. Complete remapping was observed between the square and circle in Wills et al., 2005, whereas partial remapping was observed in Leutgeb et al., 2005b. We believe that the findings of partial vs. complete remapping is the major difference in the findings of these papers, and is the one we focus on explaining.

What differences in protocols led to these differences in results? In addition to all the idiosyncrasies of individual lab protocols, there were two major explicitly described differences between their protocols. One is that they used different training protocols. Wills et al., 2005 used a training protocol designed for inducing complete remapping between square and circle in 6 days, and excluded animals that did not meet that criterion. Leutgeb et al., 2005b used a similar training as Lever et al., 2002, see Figure 3) for three weeks. The second difference was that Wills et al., 2005 presented the intermediate shapes in a scrambled order on the test day, whereas Leutgeb et al., 2005b presented the intermediate shapes sequentially based on number of sides on the test day.

The second difference (scrambled test order) was the focus of several theoretical explanations (Blumenfeld et al., 2006; Gershman et al., 2014), but a replication of Wills et al., 2005 using scrambled presentation resulted in limited remapping (Colgin et al., 2010), demonstrating that a scrambled presentation was not sufficient to force the hippocampus to use complete coherent remapping. Differences in the training protocol remain as a possible explanation. However, the problem remains that Colgin et al., 2010 attempted an exact replication of Wills et al., 2005, but got the opposite result. These differences can be seen in Figure 7A.

These results fit into a broader pattern of inconsistent results across two labs. Two experiments that led to complete remapping in the O’Keefe lab ended up leading to partial (and/or rate) remapping in the Moser lab. Training in alternating square and circle environments led to partial remapping initially and to complete remapping after 18 days in the O’Keefe lab (Lever et al., 2002), but led to partial remapping after 18 days of comparable training in the Moser lab (Leutgeb et al., 2005b). A 6 day white/morph circle-square training protocol led to complete remapping in the O’Keefe lab (Wills et al., 2005), but led to partial remapping in the Moser lab (Colgin et al., 2010). We do not believe either lab’s training to be inherently superior, but we do wish to point out that there are likely unreported idiosyncrasies of training that cause animals to consistently progress through partial remapping to global remapping more slowly in the Moser lab than in the O’Keefe lab (at least during the years 2000–2010). The main implication of this is that remapping behavior does not have a one-to-one mapping to the experimenter-defined conditions; rather, remapping behavior responds to a huge array of experiential factors, and the experimenter is only aware of a subset of these factors. Practically, this means that attempts to compare remapping behavior must be done between comparable controlled setups (as performed in the internal comparisons of Colgin et al., 2010), and comparisons should ideally not be made across labs.

To summarize, various experimental protocols for measuring remapping behavior in response to intermediate ‘morph’ environments give divergent results, which can be split into two categories: heterogeneous responses when there is only partial remapping between the extremes, and population-wide coherent responses when there is complete remapping between the extremes (Figure 7A). As we explored above (Figure 3), partial remapping and complete remapping can be observed in a single experimental protocol early and late in training, respectively. We therefore predict that both sets of results can be observed in the same lab, with the same experimental protocol, simply by presenting the intermediate ‘‘morph’’ environments early or late in training.

We show simulations of this prediction in Figure 7B. Specifically, we compute the probability that the training observations came from a single hidden state P⁢(𝐜𝟏) and the probability they came from two hidden states P⁢(𝐜𝟐) according to Equation 6. We then calculate the probability that the morph test is assigned to the same hidden state as the square assuming that the training observations came from two hidden states $P\left(c_{p\,r\,o\,b\,e}=c_{s\,q\,u\,a\,r\,e}|\mathrm{𝐜𝟐}\right)$ (Equation 8). The hypotheses that correspond to the morph being assigned the same hidden state as the square are ***S1***) that there is a single hidden state for the training and the morph is from the same state and ***S2***) that there are two hidden states for the training and the morph is from the same state as the square. The hypotheses that correspond to the morph being assigned a different hidden state than the square are ***D1***) that there is a single hidden state for the training and the morph is from a novel state and ***D2***) that there are two hidden states for the training and the morph is from the same state as the circle and ***D3***) that there are two hidden states for the training and the morph is from a novel state. We take the log posterior ratio between the ***S*** hypotheses and the ***D*** hypotheses and plot that in Figure 7B for varying number of training observations. The probability of assigning intermediate ‘‘morph’’ environments to the same hidden state as one of the extreme environments increases with the amount of training.

Thus, we suggest that a key distinction between classes of past morph results is whether there is complete or partial remapping between the extreme environments, and that complete or partial remapping can be achieved by a wide range of training protocols (as described throughout the paper) including amount of experience (as described in Figure 3).

### Population heterogeneity and rate remapping

**Figure 8.**
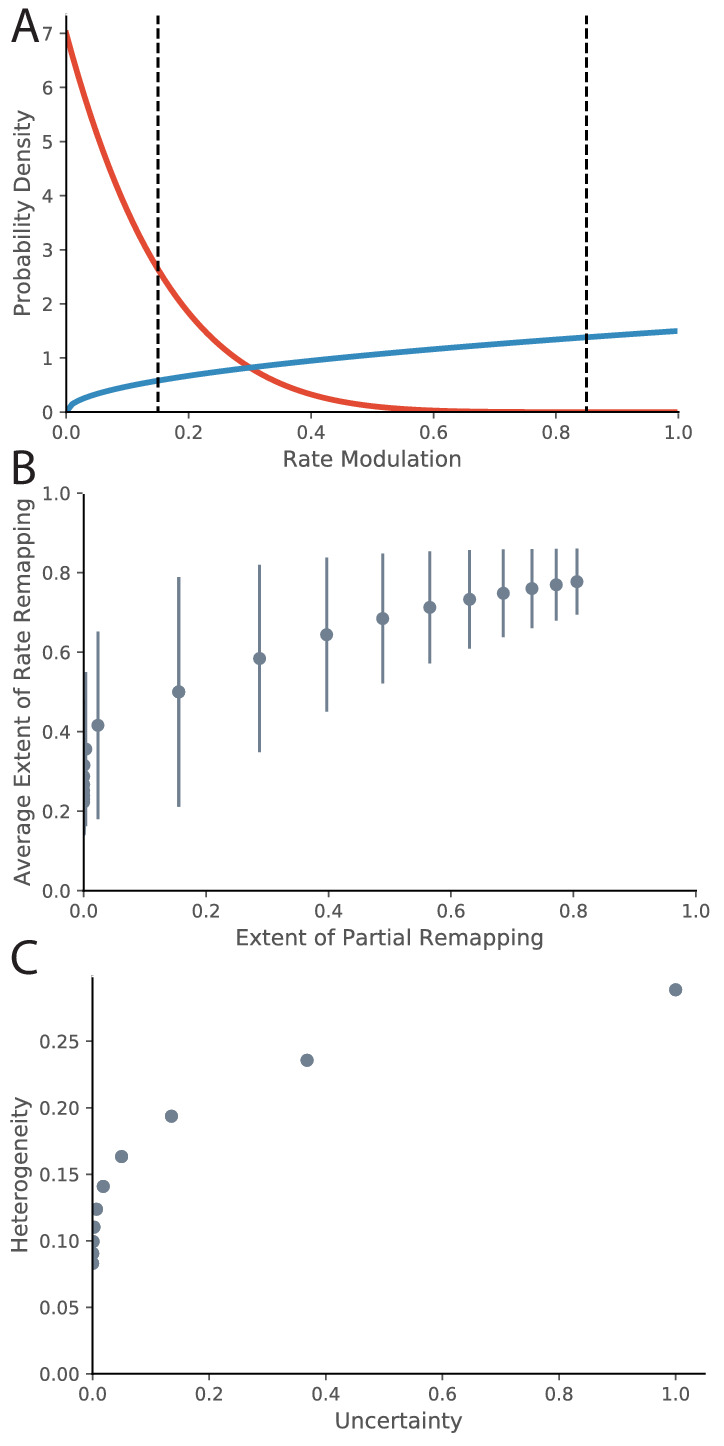
Relationship between Rate Remapping and Partial Remapping. (**A**) The Beta distribution is used to illustrate the distribution in remapping responses over the place field population. Examples of the Beta distribution for parameter values a=1,b=7 (red) and a=1.5,b=1 (blue). We can draw a correspondence between the difference between these parameters and the evidence ratio. We can also characterize remapping behavior by saying that place fields with rate modulation less than the left-hand black dotted line do not remap, place fields between the black dotted lines rate remap, and place fields with rate modulation greater than the right-hand dotted black line completely remap. The extent of partial remapping would then be the fraction of cells that completely remap (fall to the right of the right-hand dotted black line). (**B**) The average extent of rate remapping has a positive relationship with the extent of partial remapping for a range of evidence ratios. Error bars are the standard deviation of the Beta distribution for those parameter values. (**C**) The amount of heterogeneity in rate remapping extents across the population of place fields has a positive relationship with the amount of uncertainty that the animal has over a range of evidence ratios.

So far, we have drawn the correspondence between evidence ratios and ‘extent of remapping’. There are a variety of ways to empirically quantify extent of remapping over the population, including average population vector correlations, average firing rate map correlations, and average change in location of place fields (Leutgeb et al., 2005a). One important question to ask is how the extent of remapping is distributed across the population. One option is that some fraction of the place fields are perfectly retained and some fraction disappear or appear. This would be called partial remapping. Another possibility is that each place field modulates its peak firing rate. This would be called rate remapping. As mentioned in the Empirical Background section of the Introduction, both types coexist. We can qualitatively model this in the following way. For a given experimental manipulation, there is some distribution across the population of how much the firing rate is modulated. We can quantify for a given place field the extent of firing rate modulation using a measure such as 1-(lower firing rate)/(higher firing rate), ranging from 0 (identical firing in both conditions, i.e., no remapping) to 1 (place field exists in one condition but not in other condition, i.e., remapping). Intermediate values between 0 and 1 correspond to the extent of rate remapping. A common distribution with support on the interval [0,1] is the Beta distribution, which we will use for illustration. The Beta distribution has two parameters a and b, which correspond to relative probability mass on 1 and 0 respectively. We can map our evidence ratio loosely onto these parameters for illustration by saying that the value of the parameter corresponding to the preferred hypothesis is the magnitude of the evidence ratio + 1 and the value of the other parameter is 1. In this manner, the evidence ratio equals b-a. For example an evidence ratio of +6 would have a=1,b=7 because a positive evidence ratio prefers no remapping, that is more probability mass on 0 (red line in Figure 8A). This evidence ratio is at the border of our range of partial remapping. If we put thresholds at 0.15 and 0.85 (dotted lines) for indistinguishable from no remapping and complete remapping respectively, we see that 0% of place fields completely remap, 31% rate remap, and 69% do not remap, which is consistent to what we would expect for that magnitude of evidence ratio. Conversely, an evidence ratio of −0.5 would correspond to parameter values of a=1.5,b=1 (blue line in Figure 8A). This evidence ratio is near the center of our range of partial remapping, and we see that 22% of place fields completely remap, 72% rate remap, and 6% do not remap.

This framework can organize some empirical observations into patterns that we would predict would generalize. One is that the fraction of place fields that completely remap seems to be correlated with the magnitude of rate remapping that is observed across different protocols. For example, the paper that coined the term ‘rate remapping’ (Leutgeb et al., 2005a) explores several different experimental manipulations. Rank ordering of their manipulations according to their measures of rate remapping (firing rate changes and population vector correlations) matches well with the rank ordering according to their measures of complete remapping (place field correlation and center of mass change). Similar patterns of partial remapping occurring in concert with rate remapping can be observed in many other reports (Wood et al., 2000, their Figure 5, Anderson and Jeffery, 2003, their Figure 2, Lu et al., 2013, their Figure 3, Sanders et al., 2019, their Figure 1). The framework of a distribution of remapping behaviors over the place field population captures this phenomenon. If we look at parameter values ranging from a=10,b=1 to a=1,b=10, we see a positive relationship between the extent of rate remapping and the extent of partial remapping (Figure 8B). The only report we are aware of in which rate remapping is observed in the absence of partial remapping is that of Allen et al., 2012. It is notable that they observe much weaker rate changes than the conditions studied in other papers: visual inspection of their Figure 6B1 (top) shows an average rate modulation of ∼(17-14)/17 = 17%. This would roughly correspond to the distribution shown as the red line in Figure 8A which does indeed show a population of place fields that rate remap but a negligible fraction of place fields that completely remap. Overall, a comprehensive literature review suggests that extent of rate remapping is correlated with the extent of partial remapping, as would be expected if there is a distribution over the population in sensitivity to manipulations. Future experiments are needed to verify this hypothesis directly.

Another pattern that seems to occur in the literature is that increased uncertainty seems to correlate with heterogeneity in remapping behavior across the population of place fields. One example of this pattern is the difference in heterogeneity observed between the protocol of Leutgeb et al., 2005b compared to that of Wills et al., 2005. More heterogeneity was observed in the experiment of Leutgeb et al., 2005b, who were in the partial remapping regime (red line in our Figure 7B), compared to the relatively coherent response observed across the population in the experiment of Wills et al., 2005, who were in the global remapping regime (blue line in our Figure 7B). Other examples of population heterogeneity occurring with uncertainty include findings by Lee et al., 2004; Chen et al., 2013; Gothard et al., 1996. The framework of a distribution of remapping behaviors over the place field population captures this phenomenon. If we look at parameter values ranging from a=10,b=1 to a=1,b=10, we see a positive relationship between heterogeneity in response (as measured by the standard deviation of rate remapping extents across the population) and level of uncertainty (as measured by inverse exponentiated absolute value of evidence ratio). Future experiments are needed to test this hypothesis directly. Ideally, behavioral measures of uncertainty would be compared to neural measures of population heterogeneity.

### Animal-to-animal variability

One challenge in the study of hippocampal remapping is that different animals respond differently to the same environments. Indeed, many of the previously discussed studies reported significant heterogeneity across animals in remapping behavior. Studies of the development of remapping over the course of learning frequently report that different animals learn at differing rates (Bostock et al., 1991; Lever et al., 2002). In fact, the variability across animals is frequently a nuisance in running experiments. The pre-training for one of the morph experiments described above (Wills et al., 2005) had three different ways that the observations could be partitioned. Out of the six animals they trained, four animals partitioned the observations in the way the experimenters expected, and the other two animals partitioned the observations in the other two possible ways (and therefore were excluded from the rest of the study).

**Figure 9.**
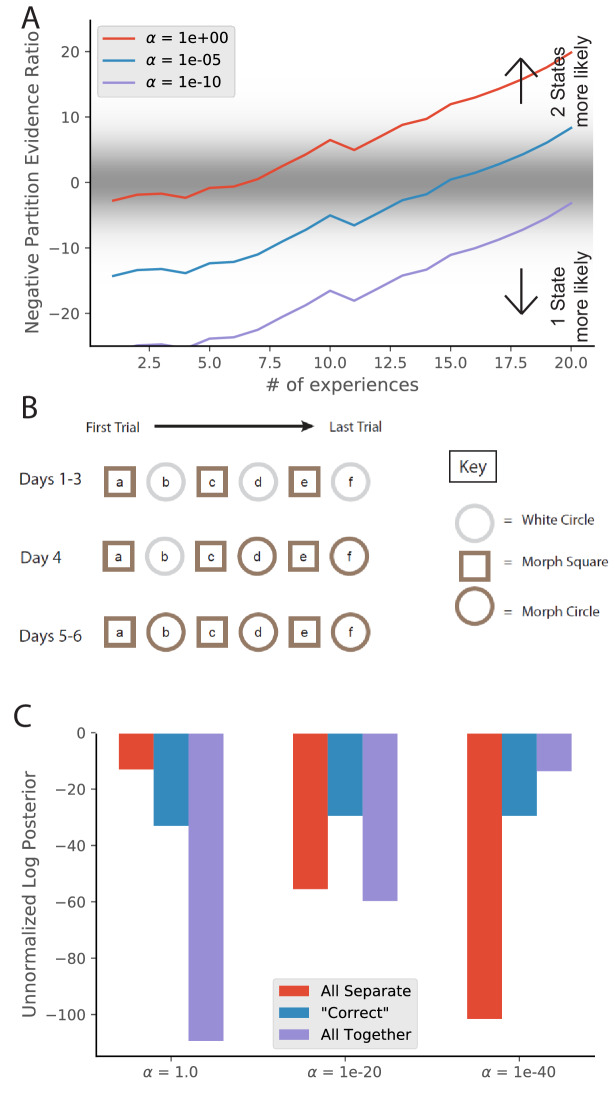
Animal-to-animal variability may be the result of animal-specific parameter settings. (**A**) Simulations from Figure 3C with different values of α. Larger values of alpha lead to a greater tendency to infer a larger number of hidden states, and therefore a faster transition from preferring the single-state model to the two-state model. (**B**) The training protocol from Supporting Figure 1C of Wills et al., 2005. (**C**) In red is the probability assigned to the hypothesis that the white circle, morph circle, and morph square are all generated by separate hidden states. In blue is the probability assigned to the hypothesis that the white circle and morph circle are generated by the same hidden state and the morph square is generated by a separate hidden state, which is the hypothesis that the authors expected. In purple is the probability assigned to the hypothesis that all of the enclosures are generated by the same hidden state (Equation 6). Different settings of α result in different preferred assignments of observations to hidden states, corresponding to the finding that different animals had different remapping behaviors.

The hidden state inference model offers one way to capture this heterogeneity across animals. The concentration parameter α (see Materials and methods) controls the tendency to infer new hidden states when unexpected data are observed. Variation in this parameter was previously used to model age-dependent (Gershman et al., 2010; Gershman et al., 2017b) and individual (Gershman and Hartley, 2015a) variability in learning. While partitioning large amounts of cleanly separated data is insensitive to changes of α over several orders of magnitude, α can have effects on partitioning of ambiguous or insufficient data. For example, if we take the learning of remapping explored in Figure 3, changes in the value of α can alter the speed at which the model switches from preferring a one-state hypothesis to a two-state hypothesis (Figure 9A). Moreover, if we take evidence ratios around 0 as indicative of partial remapping, different α values can lead to different lengths of time spent in the partial remapping regime, even for the exact same set of experiences.

To explore a second manifestation of animal variability, we ran a simulation resembling the training of Wills et al., 2005. We characterize observations with two features: shape and color of the enclosure. The white circle is characterized by a 2D Gaussian with means [1, 1], the morph circle is characterized by means [1, -1], and the morph square is characterized by means [−1,–1]; all standard deviations are 0.1. We provided the model with observations from these generative distributions according to the schedule used by Wills et al., 2005 (Figure 9B). We then asked the model to assign an unnormalized posterior probability to the following hypotheses:

1. Each of the environments were drawn from separate hidden states (Figure 9C, red bars), corresponding to ‘did not show wooden circle to morph-circle pattern transfer.’
2. The circles were the same and were different from the square (Figure 9C, blue bars), corresponding to the selection criterion adopted by Wills et al., 2005.
3. All the observations were drawn from a single hidden state (Figure 9C, purple bars), corresponding to ‘failed to show rapid remapping in the morph-square and the wooden circle'.

Different values of α lead to variation in relative preferences for these hypotheses.

These results invite the interpretation that animal variability may be understood in terms of individual differences in the α parameter (though of course other parametric variations might produce some of the same effects).

### The effect of cue variability

**Figure 10.**
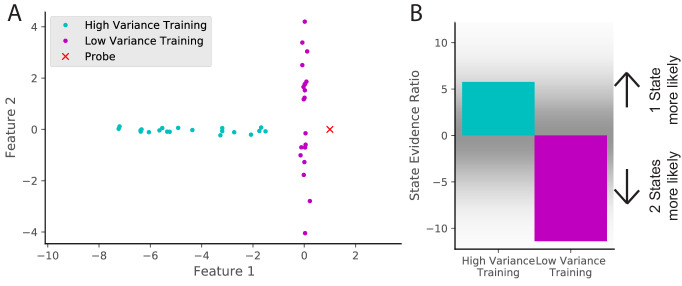
Cue variability should affect remapping behavior. (**A**) Two training protocols (cyan and magenta) give (**B**) qualitatively different hidden state inferences when presented with the same novel observation (red dot in A). The cyan training is drawn from a Gaussian with mean [−5,0] and standard deviations [2, 0.1], whereas the magenta training is drawn from a Gaussian with mean [0,0] and standard deviations [0.1, 2]. The probe is presented after 20 training observations. The state evidence ratio here is the comparison between the assignment of the probe to the same hidden state as the training samples vs. a novel hidden state (Equation 11).

In this section, we explore an experimental prediction of the model that highlights one of its key insights: remapping critically depends on past experience. Consider an environment that is characterized by two features. We can separate animals into two training groups: one in which feature one is highly variable and one in which feature two is highly variable (cyan and magenta dots in Figure 10A). We then probe with an observation that has a novel value in feature 1 (red x in Figure 10A). The model predicts that an animal trained with higher variability in feature one will be more likely to assign the novel observation to the same state as the previous observations (i.e., not to remap; Figure 10B). Intuitively, high variability will make the place fields more ‘‘tolerant’’ of deviations from the central tendency of the distribution. After initial submission of this paper, a similar result was posted as a preprint (Plitt and Giocomo, 2019).

By analogy, imagine a building with many similar conference rooms. One conference room always has its chairs arranged in a particular configuration (a low variability context), whereas another conference room frequently has different configurations (a high variability context). Intuitively, a change in the expected configuration in the low variability context will prompt the inference that you must be in a different room (and hence the place cells in your hippocampus will remap), whereas a change in the expected configuration in the high variability context will not. In the high variability context, you expect the unexpected (cf. the concept of ‘expected uncertainty’ in Yu and Dayan, 2005).

## Discussion

We have proposed that hippocampal remapping provides a window into the process of hidden state inference. According to our framework, animals receive a stream of observations (data points), which they attempt to partition according to the hypothetical hidden states that generated them. Bayesian inference offers a natural solution to this problem. The specific form of Bayesian nonparametric model that we employed here has been previously invoked to explain a number of other hippocampal-dependent behavioral phenomena (Gershman et al., 2010; Gershman et al., 2017a; Gershman et al., 2014). In this paper, we showed that this model recapitulates a broad range of remapping phenomena.

Central to our account is the idea that remapping reflects inferences about the hidden state, and in particular that partial remapping corresponds to high levels of uncertainty. Manipulations of sensory cues, environmental geometry, and training can all be understood in terms of their effects on state uncertainty. While this account has the potential to unify many phenomena under a common theoretical umbrella, there are still many limitations, loose ends and open questions, which we discuss below.

### What is the feature space?

Our model takes feature vectors as its inputs, but what are these features? In our simulations, we allowed them to be highly abstract idealizations. Ultimately, a biologically grounded theory must specify these features in terms of the inputs to the hippocampus. Furthermore, it will be necessary to more explicitly specify what timescale the model is operating on, since different features are relevant at different timescales. Although we have focused on the timescale of hours to days, map switches can occur on the subsecond timescale (Olypher et al., 2002; Jezek et al., 2011; Kelemen and Fenton, 2016).

One general hypothesis about the feature space encoded by the hippocampus is the *successor representation* theory (Stachenfeld et al., 2017), which posits that place cells encode a predictive map of the state space. On this view, the feature inputs to the hippocampus correspond to state features. This raises the intriguing possibility that remapping should be sensitive to predictive relationships between states. Many studies have observed that place cells are modulated by prospective information like the animal’s future trajectory (e.g., Battaglia et al., 2004; Ferbinteanu and Shapiro, 2003). It is less clear whether there is any evidence for global remapping as a function of changes in prospective information.

### What is an observation?

In this paper, we characterize ‘observations’ as feature vectors. A question is what qualifies as a single observation? For the experiments we highlight in the Results section, we define a single observation as an entire session, as temporal continuity constrains within-session variability in a way that between-session variability is not constrained. However, a session is not the only time scale over which an animal may perform hidden state inference. Different definitions of a single observation can highlight phenomena occurring on different time scales, such as the map switches that can occur on a timescale of 100 ms-1s (Olypher et al., 2002; Kelemen and Fenton, 2016).

One limitation is that an observation must be sufficiently long for sampling of all features of an environment. Indeed, it takes some time for the representation of an environment to settle down as the animal samples the environment (Leutgeb et al., 2004). The process of sampling the environment is a research question in its own right and the nature of that process will have important effects on the hidden state inference process we describe here. 

A related question is how often hidden state beliefs are recalculated. One possibility is that it is recalculated every theta cycle. Another is that change detection or event segmentation may be used (Franklin et al., 2019).

### Approximate inference

As discussed in the first section of the Results, exact inference over assignments of observations to hidden states is intractable, because the number of possible partitions is too large. As a result of this intractability, for most of the paper, we have limited ourselves to comparisons between a small number of hypotheses (selected based on the fact that most of the posterior probability will be concentrated on these hypotheses). This should be understood as an analytical heuristic rather than as an algorithmic theory of how the brain approximates probabilistic inference. It may be that the hippocampus does explicitly compare a small number of hypotheses. The key step in that algorithm would be generating appropriate proposals. A complete algorithmic theory must explain how the brain deals with arbitrarily large hypothesis spaces.

One idea is to model the hippocampus as stochastically sampling the hypothesis space (Fox and Prescott, 2010; Savin et al., 2014). According to this view, a sampling approximation approach would discretely represent each hypothesis with a frequency proportional to its probability. This fits nicely with the empirical finding that multiple maps can alternate rapidly (Kelemen and Fenton, 2016; Kelemen and Fenton, 2010; Jackson and Redish, 2007; Kay et al., 2019; Jezek et al., 2011). Some of these findings suggest an oscillatory implementation, whereby each theta cycle plays the role of a single sample from the distribution of possible hidden states, and the extent of map switching corresponds to the degree of uncertainty about hidden state assignment. Indeed, map switching increases at points of uncertainty (Jezek et al., 2011). We would additionally predict that measures of map switching such as overdispersion would decrease over the course of experience in protocols such as that of Lever et al., 2002 as one hypothesis dominates (i.e., as the evidence ratio between alternative hypotheses gets farther from 0 in Figure 3C).

### Priors

We have focused on examples where specification of the appropriate generative distribution has been relatively straightforward: Gaussians (or the analogous Von Mises for circular variables) have fit nicely. Not every problem corresponds to these generative distributions, however, and the animal may have to perform inference and/or meta-learning over which generative distributions are appropriate for each cue class.

Another type of prior that we use is the Chinese Restaurant Process (CRP) prior over partitions of observations into hidden states, as discussed in the Materials and methods. We explore the utility of the CRP’s α parameter in capturing animal-to-animal variability in remapping response to similar experimental protocols. The α parameter also has the potential to itself be learned. For example, animals trained with a larger number of hidden states (e.g., enriched environment) may grow to employ a larger magnitude α than animals trained with a smaller number of hidden states. As α defines the animal’s relative preference for a larger number of hidden states, modulation of the value of α in response to the complexity of past experience would be adaptive.

### Hierarchical inference

Throughout this paper, we have assumed that hidden states are independent, but in reality, hidden states can share some structure while continuing to be distinct. Hierarchical inference can be useful to solve these problems (Gershman and Niv, 2015b). Our model does not directly address the question of hierarchical inference in the hippocampus. One possibility is that hidden state inference is explicitly hierarchical even within a co-localized population. McKenzie et al., 2014 found a hierarchy of representational similarities in dorsal hippocampus. This could correspond to a single population of place cells performing hidden state inference simultaneously at different levels of a hierarchy. For example, although we have focused on hidden states corresponding to ‘context’, similar inference could be applied to identifying ‘position’ or ‘item’ categories used by McKenzie et al., 2014. Another way that the model could be extended is based on the organization of place fields by size along the dorso-ventral axis of the hippocampus. An analogy between place field sizes and hidden state inference on the level of context raises an interesting possibility. The range of locations that are categorized as the same in terms of being included in the same place field increases along the dorso-ventral axis (Maurer et al., 2005), so too the range of observations that would be categorized as the same in terms of context-level hidden state inference may increase along the dorso-ventral axis. This gradient could be implemented if the same hidden state inference process would occur independently at different distances along that axis with different values of α, leading to different proclivities for opening new hidden states. In that way, it would be possible for two observations to be assigned to the same hidden state at one location along the axis and assigned to different hidden states at another location along the axis, leading to a partial sharing of learning between the two observations. A test of this suggestion would be that remapping behavior should be different at different locations along the dorsoventral axis. More research is needed to determine how hierarchical inference is performed in the hippocampus.

### Long-term instability of place fields

Early reports of place field stability demonstrated the existence of place fields that maintained spatial preference over the period of a month (Thompson and Best, 1990). However, recent work with sufficient statistical power have painted a different picture. Large scale recordings of hundreds of place cells over the course of a month have shown that place field instability is the norm over long time periods (Ziv et al., 2013; Rubin et al., 2015). It is possible that these reports of place field instability are the result of misattribution of place cell identity when comparing across sessions, but much effort has been put into avoiding such methodological issues (Sheintuch et al., 2017). One possible way to incorporate this finding within the hidden state inference framework is to appeal to computational work that allows for maintained fidelity of representation even with changing neuronal substrate (Raman et al., 2019; Rule et al., 2019; Druckmann and Chklovskii, 2012). On the other hand, changes in neural representation may reflect the cognitive finding that memory content can actually be modified over time through processes such as memory reconsolidation (Lee et al., 2017). Another approach is to assert that drift over time in place cell activity reflects an interest in maintaining different hidden states for the same observation occurring at different points in time. The extension of our framework with a generative model that explicitly accounts for time would be fruitful (see for example Gershman et al., 2017a).

### Behavioral relevance of remapping

An interesting fact about the remapping literature is that there has been relatively little work done relating remapping to behavior. The widespread assumption in the field is that hippocampal remapping is the neurophysiological substrate for context-dependent learning (Colgin et al., 2008), and that is an assumption that we have followed here. There is some correlational evidence of a connection (Kentros et al., 2004; Kennedy and Shapiro, 2009). However, to our knowledge, there has not been a demonstration of a causal connection (Kubie et al., 2019). In fact, Jeffery et al., 2003 show task performance transfer between two conditions that show near-global remapping.

We suggest an experiment that emphasizes the role of the hippocampus in ‘latent learning’ (Tolman, 1948), and relies on our prediction that different training would give rise to different remapping behavior (Figure 10). Train two groups of animals to either remap or not remap to a given manipulation. For example, train one group of animals in a morph box where the configuration of the walls changes every day, which we would expect to lead to a lack of remapping between circle and square configurations. Train the other group with the same morph box but only presenting the square or circle configurations, similar to the training protocol of Lever et al., 2002, which we would expect to eventually lead to remapping between the circle and square configurations. Once the expected remapping behavior is neurophysiologically verified, the animals would undergo fear conditioning in one configuration (square or circle). The prediction would be that generalization of the fear memory to the other configuration would depend on which training the animal had received and correspondingly, which remapping behavior the animal had exhibited. An experiment such as that would be a first step towards ascertaining the behavioral relevance of hippocampal remapping.

### Rotation experiments

There are several classes of empirical results that are related to the results explained in this paper, but not directly explained by our model. For example, in rotation experiments, the experimenters manipulated cues associated with the environment itself (‘proximal cues’ or ‘maze cues’) and/or manipulated cues associated with the room that the recording environment was placed in (‘distal cues’ or ‘room cues’). They asked questions such as whether the place cells followed the rotation of the maze cues or the room cues (Shapiro et al., 1997), and whether the place cells followed the animal’s own motion or the motion of the cues (Knierim et al., 1998). The answers to these questions were generally inconclusive, as they were sensitive to slight differences in protocol across labs. However, a consistent finding was that the results changed over the course of experience. For example, when a cue was repeatedly moved relative to other cues in an unstructured way, the cue lost control over the rotational alignment of the place fields (Knierim et al., 1995). While we do not explicitly model spatial relationships in our simulations, Knierim’s finding is similar to the training variance effect described in Figure 10: when the model is trained with observations for which a cue has high variance, further variation in that cue is less likely to cause a new hidden state to be inferred.

Conversely, in Shapiro et al., 1997, the maze cues and room cues were each rotated 90 degrees in opposite directions. Initially, the place cell representation split, some following room cues and some following maze cues. However, after a few repetitions, the place cell representation remapped between the two conditions. This is reminiscent of the simulations in Figure 3, where a particular cue manipulation (square-circle) initially does not cause remapping, but after sufficient repetition, the place cells remap between the conditions; more evidence has been gathered to support the hypothesis that two distinct hidden states exist.

### Types of remapping

An influential interpretation of the literature has been that there are two main types of remapping: ‘global remapping’ and ‘rate remapping’. In particular, it has been argued that global remapping corresponds to changes in physical location whereas rate remapping corresponds to changes in condition that occur at those locations (Leutgeb et al., 2005a; Colgin et al., 2010; Alme et al., 2014; Lisman et al., 2017). As discussed in the Introduction, the lines between global remapping and rate remapping are not so sharp. Global remapping can occur between conditions at the same physical location (Wills et al., 2005), and rate remapping can occur between different physical locations (Spiers et al., 2015). Moreover, the same manipulation can cause global remapping or rate remapping at different points in training (Lever et al., 2002). Our work provides an explanation for why there are not clear delineations of which manipulations cause which types of remapping. The animal must *infer* hidden states from its observations. Alternative hypotheses must be considered as long as ambiguity exists about the appropriate assignment of observations to hidden states. This uncertainty about hidden state assignment can manifest as ‘partial’ or ‘rate’ remapping. The statistics of these hidden states can be learned over the course of experience, leading to increased certainty about hidden state assignments. This increased certainty can be observed as more definitive ‘global’ remapping or conversely, lack of remapping. One of our key points is that these categories are better thought of as existing along a continuum defined by state uncertainty.

### Relationship to other theories

How does this proposal relate to other theoretical perspectives on hippocampal remapping? We can contrast our model with a basic similarity threshold model, according to which each state is associated with a fixed set of features, and new observations would be classified as the same or different based on whether they exceed some threshold of change detection. This model does not capture some of the key phenomena associated with remapping; in particular, it cannot account for any of the ways in which learning affects remapping.

One major model of remapping is the attractor network. Based on early work by Hopfield, 1982, the idea is that activity patterns associated with particular observations are learned by the network so as to be able to recover those activity patterns when degraded versions are presented. One attractor network implementation that has been specifically used to model remapping results was proposed by Blumenfeld et al., 2006. They sought to explain the difference in results between Wills et al., 2005 and Leutgeb et al., 2005b by focusing on the scrambled order of the morph sequence. Their model was a conventional Hopfield network augmented with a ‘weight’ term to change the pattern strength based on the novelty of that pattern. This led to attractors that were lumped together when the morph experiences were presented in sequential order instead of in a scrambled order. However, later work Colgin et al., 2010 demonstrated that the order of presentation of the morph experiences was not the decisive factor in the qualitative results of the morph experiments (as described in more detail in the ‘Morph Experiments’ section of the Results).

The attractor network perspective can be connected to our hidden state inference model by examining the probabilistic version of the Hopfield network, known as the *Boltzmann machine* (Ackley et al., 1985). The basins of attraction can be understood heuristically as feature configurations for distinct hidden states. One can make this heuristic connection more precise by defining an explicitly state-dependent energy function combined with a distribution over states, which would correspond to a mixture of Boltzmann machines (Nair and Hinton, 2009; Salakhutdinov et al., 2013).

In computational neuroscience, attractor networks are usually used as mechanistic descriptions of neuronal dynamics, unlike our hidden state inference model that operates at a higher level of abstraction. Thus, comparison of the two approaches is not entirely straightforward. It is possible that an attractor network could be used as an implementation of parts of the hidden state inference model. For example, inference about new states vs. old states is conceptually similar to the distinction between ‘‘pattern separation’’ in the dentate gyrus and ‘‘pattern completion’’ in CA3 (Knierim and Neunuebel, 2016; Rolls and Kesner, 2006). The attractor network describes *how* pattern separation and completion work. The hidden state inference model describes *why* pattern separation and completion work the way they do.

Our hidden state inference model is similar in spirit to the probabilistic model of remapping developed by Fuhs and Touretzky, 2007. In that model, each context is represented by a Hidden Markov Model. Remapping is then formalized as a model comparison problem. Like our model, their calculation weighs both simplicity of a hypothetical partition and its fit with the observed data. They use their model to explain gradual remapping (Lever et al., 2002), failure to generalize (Hayman et al., 2003), and some aspects of reversal learning and sequence learning. The technical differences between our models are subtle and do not change the general conclusions, which we share. Our work can be thought of as an update to the work of Fuhs and Touretzky, 2007. The main addition of this work is to stress the role of uncertainty and its relationship to partial remapping, rate remapping, and population heterogeneity, relationships that are highlighted in Figures 2, 4, 7 and 8, for example.

The Temporal Context Model (TCM, Howard and Kahana, 2002) was originally motivated by human episodic memory, but has also been applied to hippocampal/entorhinal recordings (Howard et al., 2005). TCM defines a temporal context, which is a filtered version of the observations preceding the current observation. Later observations can be used to recall the temporal context of earlier observations. This model is particularly well suited to capturing hippocampal response to temporally modulated observations, such as those we explore in Figure 5 (Markus et al., 1995). Analogies can be drawn between TCM and our account (see Gershman et al., 2017a, for discussion) by drawing a correspondence between temporal context and hidden state. The process of retrieving a past temporal context on presentation of a novel observation would be analogous to assigning that novel observation to the same hidden state as the previous observations with the same temporal context. One difference is that recall of temporal context is strictly similarity-based whereas hidden state inference explicitly models the generative distribution and therefore would have different predictions in cases that depend on training variance such as Figure 10.

There has been discussion about the role that context plays in context-dependent learning (Kubie et al., 2019). One approach is to consider context to be a cue that can acquire associations and competes with other cues (Rescorla and Wagner, 1972; Grau and Rescorla, 1984). Another is that context modifies the associations that are learned with other cues (Bouton, 1993; Nadel and Willner, 1980; Hirsh, 1974). We have previously proposed a model in which the animal performs inference over the alternative causal structures of the environment (Gershman, 2017). Context can play each of the previously mentioned roles depending on the previous training that the animal received. We would postulate that the hidden state calculated through the process outlined in this paper would be used as an input to the inference described by Gershman et al., 2017b.

### Conclusion

Place field remapping has long been one of the most puzzling aspects of hippocampal physiology, yet still lacks a comprehensive theoretical account. In this paper, we have taken steps towards such an account, starting with a normative formulation of the problem that we believe remapping is solving, namely hidden state inference. The algorithmic and biological underpinnings of this theory remain incomplete, setting a clear agenda for future theoretical work.

## Materials and methods

### Generative model

We model the animal’s sensory inputs (observations) as a vector $𝐲=\left[y_{1},\ldots ,y_{D}\right]$ consisting of D features. The specific representation of these features varies across experimental paradigms. The animal assumes that observations are generated by discrete hidden states. At each time point, a state is stochastically selected according to prior P⁢(c), and the observation features are sampled from the observation distribution associated with that state, $P\,\left(𝐲|c,\theta_{c}\right)$, where $\theta_{c}$ represents the parameters of the observation distribution for state c. For notational simplicity we will omit the time index t whenever it is unnecessary for the exposition.

We place a prior $P\,\left(\theta_{c}\right)$ over the parameters and then marginalize to obtain the likelihood that a set of m observations ${𝐘}_{c}=\left[{𝐲}_{1},\ldots ,{𝐲}_{t},\ldots ,{𝐲}_{m}\right]$ came from a single state c

(2) $$P(Y_{c}|c)=\int_{\theta_{c}}\left[\prod_{t=1}^{m}P(y_{t}|c,\theta_{c})P(\theta_{c})\right]d\theta_{c}$$

which we can extend to obtain the likelihood that a set of observations 𝐘 came from a set of K hidden states 𝐜

(3) $$P\,\left(𝐘|𝐜\right)=\prod_{k=1}^{K}P\,\left({𝐘}_{c_{k}}|c_{k}\right)$$

We model real-valued features with a multivariate normal observation distribution. The parameter vector is given by $\theta_{c}=\left(\mu_{c},\Lambda_{c}\right)$, where $\mu_{c}$ is the mean vector, and $\Lambda_{c}$ is the covariance matrix. We place a conjugate normal-Wishart distribution over these parameters (see Murphy, 2007 for more details), with hyperparameter values $\mu_{0}=0$ (prior mean), $\kappa_{0}=0.001$ (scale parameter), $\nu_{0}=0.02$ (degrees of freedom), and $T_{0}=0.02*I$ (scale matrix), where I is the D-dimensional identity matrix.

We model circular variables with a Von Mises observation distribution and a normal-gamma prior over the parameters. The hyperparamters of the prior are given by: $\mu_{0}=0$ (prior mean), $\kappa_{0}=0.001$ (scale parameter), $\alpha_{0}=0.01$ (shape parameter), and $\beta_{0}=0.01$ (rate parameter). Because in this case we cannot marginalize over parameters analytically, we used numerical integration.

To motivate our prior over hidden states, we start with a few basic desiderata: (i) the prior should be defined over an unbounded state space, allowing new states to be continually created; and (ii) the prior should prefer a small number of states, to facilitate generalization across observations (a form of Occam’s razor). These assumptions are satisfied by a simple nonparametric distribution known as the *Chinese restaurant process* (CRP; Aldous, 1985; Gershman and Blei, 2012a), which samples states according to the following sequential process:

(4) $$P(c_{t}=k|c_{1:t-1})=\left\{ \begin{array}{ll} \frac{m_{k}}{t-1+\alpha } & if\;\;k\leq K\\ \frac{\alpha }{t-1+\alpha } & if\;\;k=K+1 \end{array}\right.$$

where $m_{k}$ is the number of previous observations assigned to state k, K is the total number of states created prior to time point t, and α≥0 is a concentration parameter that controls the propensity to create new states. When α=0, all observations will be generated by the same state. As α approaches infinity, each observations will be generated by a unique state. More generally, the expected number of states after N observations is α⁢log⁡N. Another way of using the CRP prior is to analytically calculate an unnormalized log probability for a list of hidden state assignments 𝐜:

(5) $$logP(c)=K*log(\alpha )+\sum_{k=1}^{K}log\left(\Gamma \left(m_{k}\right)\right)+log\left(\Gamma \left(\alpha \right)\right)-log\left(\Gamma \left(T+\alpha \right)\right)$$

We set α=0.1 for all figures except Figure 9, in which we explicitly explore the effects of variation in α. We emphasize that using this prior does not mean that the world actually generates hidden states through this process; it simply means that we are imputing this to the animal as its *internal model* of the world.

### Inference

To compute the posterior over hidden states, the likelihood is combined with a prior over state assignments, P⁢(c), according to Bayes’ rule (Equation 1). Because we are typically dealing with a set of observations, and hence a combinatorial space of state partitions (i.e., all possible assignments of observations to states), exact inference is intractable. However, because we are generally only interested in a small number of ‘plausible’ partitions, we can simplify the problem by only assessing the relative probability of those states. The probability of each of those partitions c given a set of observations Y

(6) P⁢(𝐜|𝐘)∝P⁢(𝐘|𝐜)⁢P⁢(𝐜)

In particular, most of our simulations concern the question of whether two or three sets of observations are assigned to the same or different states. If we assume that all other partitions have probability close to 0, then we can ignore them without too much loss in accuracy. We use Equation 6 in Figure 9C.

The partition evidence ratio reported in the main text is the log odds ratio between the posterior probabilities of two hypotheses (partitions 𝐜 and ${𝐜}^{\prime }$):

(7) $$partition\;\;evidence\;\;ratio=log\frac{P(Y|c)P\left(c\right)}{P(Y|c^{\prime })P(c^{\prime })}$$

where 𝐘 denotes the set of observations, P(Y|c) is given by Equation 3 and P⁢(𝐜) is given by Equation 5. We use Equation 7 in Figures 3C, 4D, 5C and 9A.

In some cases, we are interested in computing the posterior probability that a new observation $y_{t+1}$ is assigned to a particular state conditional on a hypothetical assignment of all past observations:

(8) $$P\,\left(c_{t+1}|y_{t+1},{𝐘}_{1:t},{𝐜}_{1:t}\right)\propto P\,\left(y_{t+1}|c_{t+1},{𝐘}_{1:t},{𝐜}_{1:t}\right)\,P\,\left(c_{t+1}|{𝐜}_{1:t}\right)$$

where $P\,\left(c_{t+1}|{𝐜}_{1:t}\right)$ is from Equation 4 and

(9) $$P\,\left(y_{t+1}|c_{t+1},{𝐘}_{1:t},{𝐜}_{1:t}\right)=P\,\left(y_{t+1}|{𝐘}_{c_{k}}\right)$$

is the posterior predictive distribution characterizing the probability of observing a value of $y_{t+1}$ generated by a given hidden state $c_{k}$ given all previous observations ${𝐘}_{c_{k}}$ with that hidden state assignment. For a Multivariate Normal likelihood function with a normal-Wishart prior, this is given by:

(10) $$P(y_{t+1}|Y_{c_{k}})=gent_{v_{m_{k}}-d+1}\left(\begin{array}{l} \mu_{m_{k}},\frac{T_{m_{k}}(\kappa_{m_{k}}+1)}{\kappa_{m_{k}}(v_{m_{k}}-d+1)} \end{array}\right)$$

where g⁢e⁢n⁢t is the generalized Student-t distribution with hyperparameters $\nu_{m_{k}}=\nu_{0}+m_{k}$, $\mu_{m_{k}}=\frac{\kappa_{0}\mu_{0}+m_{k}{\bar{Y}}_{c_{k}}}{\kappa_{0}+m_{k}}$, $T_{m_{k}}=T_{0}+m_{k}cov(Y_{c_{k}})+\frac{\kappa_{0}m_{k}}{\kappa_{0}+m_{k}}(\mu_{0}-{\bar{Y}}_{c_{k}})(\mu_{0}-{\bar{Y}}_{c_{k}}{)}^{T}$, $\kappa_{m_{k}}=\kappa_{0}+m_{k}$ as discussed in Section 8.3 of Murphy, 2007.

The state evidence ratio reported in the main text is the log odds ratio between the posterior probabilities of two state assignments c and $c^{\prime }$ for a given observation $y_{t+1}$ given past state assignments ${𝐜}_{1:t}$ for past observations ${𝐘}_{1:t}$

(11) $$state\;\;evidence\;\;ratio=\log\frac{P(y_{t+1}|c,Y_{1:t},c_{1:t})P(c|c_{1:t})}{P(y_{t+1}|c^{\prime },Y_{1:t},c_{1:t})P(c^{\prime }|c_{1:t})}$$

We use Equation 11 in Figures 1B–C, 2, 4B and 10B.

### Code

All code necessary to generate all figures can be found at https://github.com/HoniSanders/Sanders-et-al-2020-Elife (copy archived at https://github.com/elifesciences-publications/Sanders-et-al-2020-Elife; Sanders, 2020).

## Data Availability

No data were analyzed in this work.

## Additional files

Transparent reporting form
